# Supplementary material for: TARM1 contributes to development of arthritis by activating dendritic cells through recognition of collagens
Source: Nat Commun. 2021 Jan 4;12:94. doi: 10.1038/s41467-020-20307-9 (PMC7782728; doi:10.1038/s41467-020-20307-9)
Supplement: Supplementary file 1 — Supplementary Information [file 41467_2020_20307_MOESM1_ESM.pdf]

Supplementary information

TARM1 contributes to development of arthritis by activating dendritic cells through recognition of collagens

Rikio Yabe, Soo-Hyun Chung, Masanori A Murayama, Sachiko Kubo, Kenji Shimizu, Yukiko Akahori, Takumi Maruhashi, Akimasa Seno, Tomonori Kaifu, Shinobu Saijo and Yoichiro Iwakura

Supplementary Table 1. PCR primers used for cloning and screening.

Name	Sequence
For 5’ arm preparation	
(sense)	ATTCCGCGGGGGTTCCCTTTCCTTGAAATTTCC
(antisense)	ATTGCGGCCGCCGGCTGGTCCAAAGGTCTTC
For 3’ arm preparation	
(sense)	ATTATCGATGTAAGTTATTCAGGGTGGGACACTCAG
(antisense)	ATTGTCGACTCATTCTGTCGTAGGTCGGTGAAAT
For ES cell screening	
(sense)	CCTTTAAGAGGAAGAACCCCAGGGC
(antisense)	CTGAACTTGTGGCCGTTTACGTCG
For 5 ’probe preparation	
(sense)	CTGTCGGAGTCCCATCTT
(antisense)	AATGTGGTCTCGGGTCC
For 3’ probe preparation	
(sense)	GGCGGACACTACACCTG
(antisense)	TCATTCTTGTTCATGTGGC
For genotyping PCR	
(sense)	TAAACCCAGCAAGCCCAGTGTTAGG
(antisense)	GGAGGCAGAGAAGGGAAAGGAGC
(antisense)	CTGAACTTGTGGCCGTTTACGTCG
For full-length Tarm1 preparation	
(sense)	ATAGCTAGCATGATCTCTAGGCTCCTTTCC
(antisense)	ATAGATATCCCAGGGTTTATTTGGAGACA
For Tarm1 ECD preparation	
(sense)	ATAGAGATATCAGAAAATGGGTCTCCTCCC
(antisense)	ATACCATGGAATCCACAGTGTAGCCTTCTGT

Supplementary Table 2. Reagents used for flow cytometry experiments.

Name	Clone	Manufacturer	Cat#	Lot#	Dilution
APC-conjugated anti-CD3e	145-2C11	Biolegend, USA	100312	B304830	1:200
APC/Cy7-conjugated anti-CD3e	145-2C11	Biolegend, USA	100330	B315704	1:200
FITC-conjugated anti-CD3e	145-2C11	Biolegend, USA	100305	B156723	1:200
Pacific blue-conjugated anti-CD3e	145-2C11	Biolegend, USA	100334	B308532	1:200
FITC-conjugated anti-CD4	RM4-5	Biolegend, USA	100406	B138112	1:200
PE-conjugated anti-CD4	RM4-5	Biolegend, USA	100407	B124811	1:200
APC-conjugated anti-CD4	RM4-5	Biolegend, USA	100412	B128970	1:200
PE/Cy7-conjugated anti-CD4	RM4-5	Biolegend, USA	100421	B213291	1:200
Pacific blue-conjugated anti-CD4	RM4-5	Biolegend, USA	100531	B255834	1:200
PE-conjugated anti-CD8	53-6.7	Biolegend, USA	100707	B132669	1:200
PE/Cy7-conjugated anti-CD8	53-6.7	Biolegend, USA	100721	B182515	1:200
APC/Cy7-conjugated anti-CD8	53-6.7	Biolegend, USA	100714	B191998	1:200
APC-conjugated anti-CD11b	M1/70	Biolegend, USA	101212	B226978	1:200
PE-conjugated anti-CD11b	M1/70	Biolegend, USA	101207	B172191	1:200
PE/Cy7-conjugated anti-CD11b	M1/70	Biolegend, USA	101226	B213161	1:200
Brilliant Violet 421-conjugated anti-CD11b	M1/70	Biolegend, USA	101235	B212918	1:200
PE/Cy7-conjugated anti-CD11c	N418	Biolegend, USA	117318	B222652	1:200
APC-conjugated anti-CD11c	N418	Biolegend, USA	117309	B256603	1:200
Brilliant Violet 421-conjugated anti-CD11c	N418	Biolegend, USA	117329	B284677	1:200
APC-conjugated anti-CD19	6D5	Biolegend, USA	115511	B284256	1:200
PE/Cy7-conjugated anti-CD24	M1/69	Biolegend, USA	101822	B179752	1:200
PE-conjugated anti-CD44	IM7	eBioscience, USA	12-0441-82	E01239-1630	1:200
PE-conjugated anti-B220	RA3-6B2	Biolegend, USA	103207	B133115	1:200
APC-conjugated anti-B220	RA3-6B2	Biolegend, USA	103212	B208579	1:200
PE-conjugated anti-CD80	16-10A1	Biolegend, USA	104707	B216170	1:200
APC-conjugated anti-CD86	GL1	Biolegend, USA	105012	B220415	1:200
FITC-conjugated anti-F4/80	BM8	Biolegend, USA	123108	B222019	1:200
APC-conjugated anti-Ly6C	HK1.4	Biolegend, USA	128015	B234315	1:200
APC/Cy7-conjugated anti-Ly6C	HK1.4	Biolegend, USA	128026	B184386	1:200
PE/Cy7-conjugated anti-Ly6G	1A8	BD Biosciences, USA	560601	3217872	1:200
Pacific blue-conjugated anti-Ly6G	1A8	Biolegend, USA	127612	B200367	1:200
APC/Cy7-conjugated anti-I-A/I-E	M5/114.15.2	Biolegend, USA	107628	B204231	1:10,000
PE-conjugated anti-Foxp3	FJK-16s	eBioscience, USA	12-5773-82	E01764-1639	1:200
APC-conjugated anti-IFN-γ	XMG1.2	Biolegend, USA	505810	B319622	1:200
Pacific blue-conjugated anti-IL-17	TC11-18H10.1	Biolegend, USA	506917	B145597	1:200
PE-conjugated anti-human IgG Fc	HP6017	Biolegend, USA	409304	B318295	5 μg/ml
FITC-conjugated anti-human IgG Fc		Jackson ImmunoResearch, USA	109-095-098	130860	5 μg/ml
Anti-mouse type I collagen	8D4A1	Chondrex, USA	7041	120442	1, 5 μg/ml
Anti-mouse type II collagen	2B1.5	ThermoFisher, USA	MA5-12789	VA2926216A	1, 5 μg/ml
Rat IgG2a isotype control antibody	RTK2758	Biolegend, USA	400501	B318085	1, 5 μg/ml
Mouse IgG control antibody		Santa Cruz, USA	sc-2025	D1416	1, 5 μg/ml
AlexaFluor647-conjugated anti-rat IgG	Poly4054	Biolegend, USA	405416	B177346	1, 2.5 μg/ml
FITC-conjugated anti-mouse IgG	Poly4053	Biolegend, USA	405305	B208134	1, 2.5 μg/ml
Biotin-conjugated anti-Ter119	TER-119	BD Pharmingen, USA	553672	2131929	2 μg/ml
Biotin-conjugated anti-B220	RA3-6B2	Biolegend, USA	103204	B129066	2 μg/ml

Supplementary Table 3. Primers for qPCR analysis.

Name	Forward	Reverse
Tarm1	TCTCTAGGCTCCTTTCCCTT	GTCACGTGGCTCTTGGT
Actb	CAATAGTGATGACCTGGCCGT	AGAGGGAAATCGTGCGTGAC
Gapdh	TTCACCACCATGGAGAAGGC	GGCATGGACTGTGGTCATGA
Tnf	GCCTCCCTCTCATCAGTTCT	CACTTGGTGGTTTGCTACGA
Il6	GAGGATACCACTCCCAACAGACC	AAGTGCATCATCGTTGTTCATACA
Il1b	CAACCAACAAGTGATATTCTCCATG	GATCCACACTCTCCAGCTGCA
Il10	GTGGAGCAGGTGAAGAGTGATTT	TCCCTGGATCAGATTTAGAGAGC
Il17a	TTTAACTCCCTTGGCGCAAAA	CTTTCCCTCCGCATTGACAC
Nfatc1	GCCAAGTACCAGCTTTCCAG	AGGGTCGAGGTGACACTAGG
Acp5	CAGCAGCCCAAAATGCCT	TTTTGAGCCAGGACAGCTGA
Ctsk	GGAGAAGACTCACCAGAAGC	GGAGAAGACTCACCAGAAGC
Dcstamp	TTGCCGCTGTGGACTATCTG	GAATGCAGCTCGGTTCAAAC
Oscar	CCTAGCCTCATACCCCCAG	CAAACCGCCAGGCAGATTG
Col1a1	GGTGCCCCCGGTCTTCAG	AGGGCCAGGGGGTCCAGCATTTC
Col2a1	GGGAATGTCCTCTGCGATGAC	GAAGGGGATCTCGGGGTTG
Mpo	ATCACGGCCTCCCAGGATAC	CCACTGTGCTAGGCTGTGGAA
Ccl2	GTTGGCTCAGCCAGATGCA	AGCCTACTCATTGGGATCATCTTG
Ccl3	TGAGAGTCTTGGAGGCAGCGA	TGTGGGTACTTGGCAGCAAACA
Ccl4	AACAACATGAAGCTCTGCGT	AGAAACAGCAGGAAGTGGGA
Ccl5	GCAAGTGCTCCAATCTTGCA	CTTCTCTGGGTTGGCACACA
Ccl19	ATGCGGAAGACTGCTGCC	AGCGGAAGGCTTTCACGAT
Ccl20	CCAAGTCTTCTCAGCGCCAT	GAATCTTCCGGCTGTAGGAGAAG
Ccl21	CCCCGGCTGCAGGAA	TGTTCAGTTCTCTTGCAGCCC
Cxcl1	AGCCACACTCAAGAATGGTC	GCCATCAGAGCAGTCTGTC
Cxcl2	CACTGCGCCCAGACAGAAGTC	TCCTCCTTTCCAGGTCAGTTATCC
Cxcl5	CATCCCCAGCGGTTCCA	CGTGAACAGCAACAGAAATGC
Cxcl9	TGCACGATGCTCCTGCA	AGGTCTTTGAGGGATTTGTAGTGG
Cxcl10	GACGGTCCGCTGCAACTG	GCTTCCCTATGGCCCTCATT
Cxcl12	GCTCCTCGACAGATGCCTTG	GACCCTGGCACTGAACTGGA
Cxcl13	CATAGATCGGATTCAAGTTACGCC	TCTTGGTCCAGATCACAACTTCA
Cxcl16	AGCACACCAGCTTGGGTACC	CATGGCTGCAGTGAGGAAGA

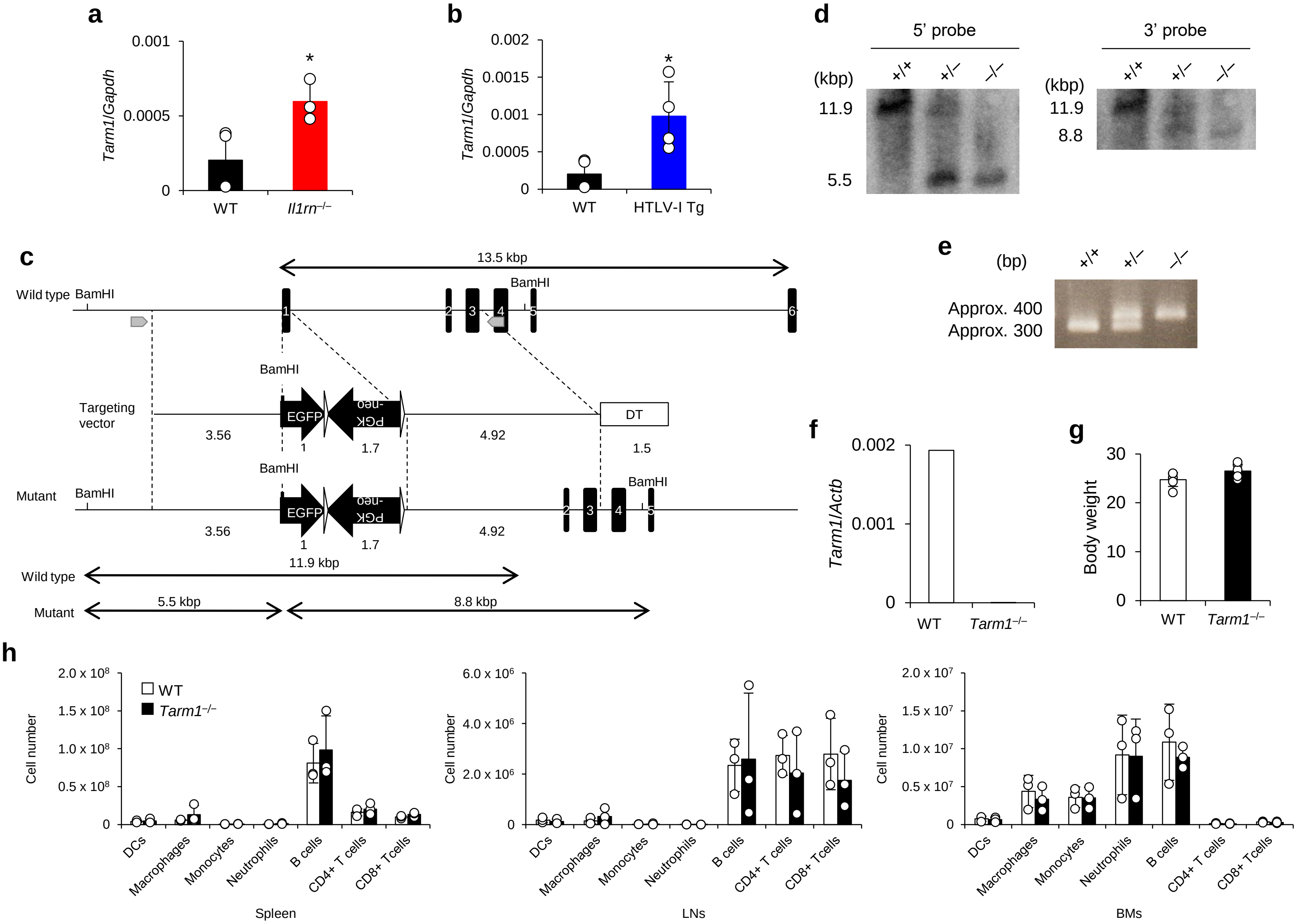

Supplementary Figure 1. Generation of *Tarm1*$^{-/-}$ mice.

(a, b) Expression of *Tarm1* in joints (knee, ankle and tarsal) of WT, *Il1rn*$^{-/-}$ and HTLV-I Tg mice was analyzed by qPCR. WT = 4, *Il1rn*$^{-/-}$ = 3 **(a)**. WT = 4, HTLV-I Tg = 4 **(b)**. Mean ± SD. *, $P < 0.05$ (two-tailed unpaired Student's *t*-test). **(c)** Generation of *Tarm1*$^{-/-}$ mice. Exon 1 was replaced by an EGFP-Neo cassette. Exons are represented by black boxes. Probe positions for Southern blotting hybridization are indicated as gray arrow boxes. **(d)** Southern blot analysis of WT (+/+), heterozygous (+/–) and homozygous mutant (–/–) mice. Genomic DNA extracted from a mouse tail was digested by *Bam*HI, and was subjected to agarose-gel electrophoresis followed by membrane transfer. The targeting bands were analyzed by Southern blotting hybridization with 5' and 3' probes. Data are representative of two-independent experiments. **(e)** Genomic PCR analysis of deleted allele. Genomic DNA isolated from a mouse tail was amplified by PCR. Data are representative of two-independent experiments. **(f)** Expression of *Tarm1* in BM cells from WT and homozygous mice was analyzed by qPCR. Data are representative of two-independent experiments. **(g)** Body weight of male littermates (8-week-old; +/+ and –/–, n = 6 each) were analyzed. Mean ±SD. (two-tailed unpaired Student's *t*-test). **(h)** The proportions of DCs ($CD11c^+F4/80^-$), macrophages ($F4/80^+$), Monocytes ($CD11b^+Ly6C^+$), neutrophils ($CD11b^+Ly6G^+$), B cells ($B220^+$), $CD4^+$ T cells ($CD4^+CD3^+$) and $CD8^+$ T ($CD8^+CD3^+$) in the spleen, LNs (brachium, axilla and groin), and BMs (femur) from WT and *Tarm1*$^{-/-}$ mice under the physiological conditions were analyzed by flow cytometry. WT = 3, *Tarm1*$^{-/-}$ = 3. Mean ± SD.

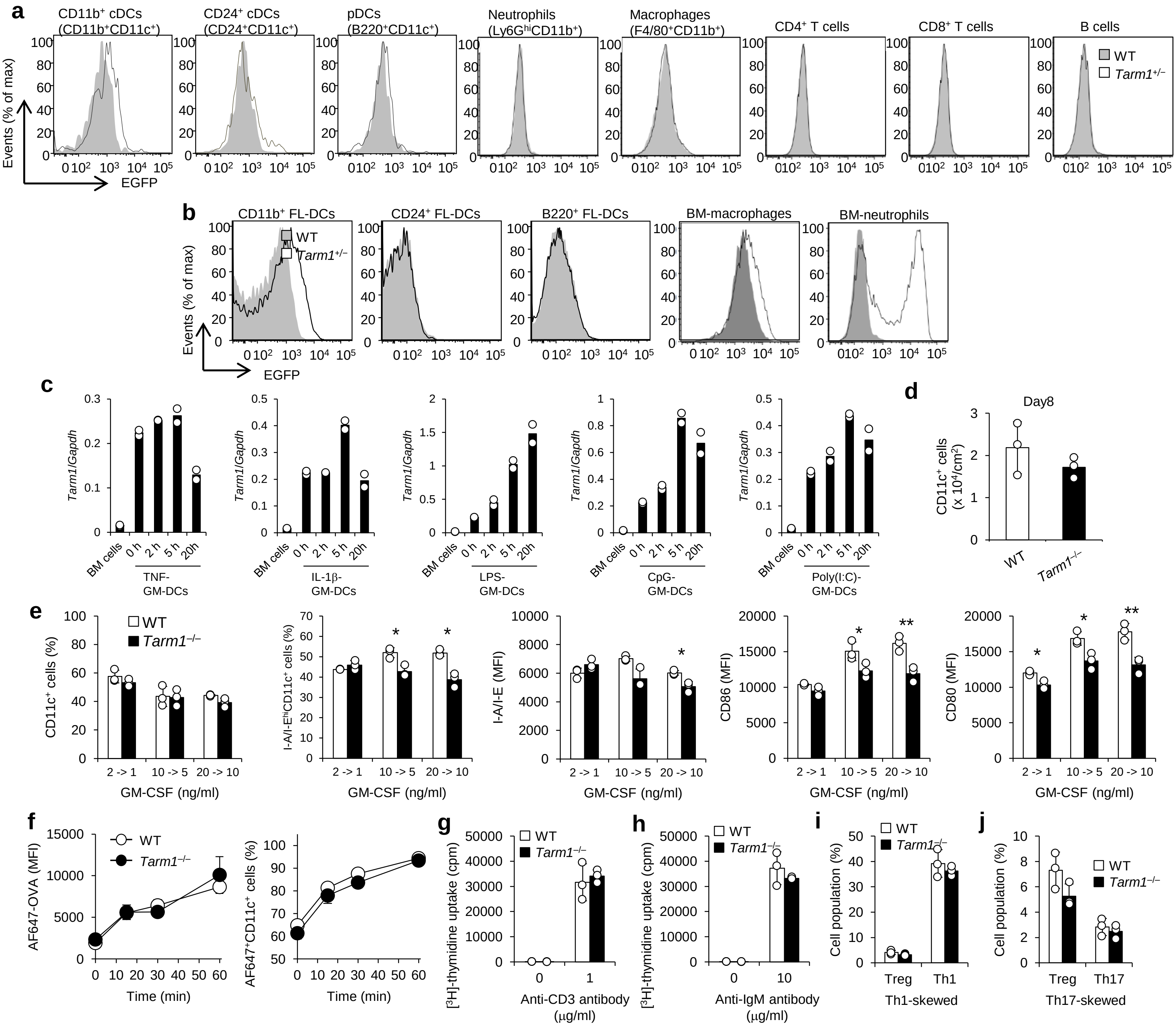

Supplementary Figure 2. Characterization of *Tarm1*$^{–/–}$ DCs.

(a) EGFP expression was analyzed in cDCs (CD11b$^+$CD11c$^+$ and CD24$^+$CD11c$^+$), pDCs (B220$^+$CD11c$^+$), neutrophils (Ly6G$^+$CD11b$^+$), macrophages (F4/80$^+$CD11b$^+$), CD4$^+$, CD8$^+$ T cells (CD4$^+$CD3$^+$ and CD8$^+$CD3$^+$) and B cells (B220$^+$) from untreated WT and *Tarm1*$^{+/–}$ mouse LNs by flow cytometry. Data are representative of three-independent experiments. **(b)** EGFP expression was examined in FL-DCs (CD11b$^+$CD11c$^+$ cells; CD24$^+$CD11c$^+$ cells; B220$^+$CD11c$^+$ cells), BM-macrophages (F4/80$^+$CD11b$^+$) and BM-neutrophils (Ly6G$^+$CD11b$^+$) from *Tarm1*$^{+/–}$ mice by flow cytometry. Non-Tg WT mice were used as controls. Data are representative of three independent experiments. **(c)** *Tarm1* expression in GM-DCs was examined after stimulation with TNF, IL-1β, LPS, CpG and poly(I:C) by qPCR at indicated times. Expression of *Tarm1* was normalized to that of *Gapdh*. Data are shown as means of duplicate wells and are representative of two-independent experiments. **(d)** The number of CD11c$^+$ GM-DCs were measured after treatment with GM-CSF by flow cytometry at day 8. Data are representative of two independent experiments. Means ± SD of triplicate wells. (two-tailed unpaired Student's *t*-test). **(e)** BM cells from WT and *Tarm1*$^{–/–}$ mice were cultivated in the presence of GM-CSF (initial concentration; 2, 10 and 20 ng/ml). The proportions of CD11c$^+$ cells and I-A/I-E^{hi}CD11c$^+$ cells and expression of activation markers, I-A/I-E, CD86, and CD80, in CD11c$^+$ cells were analyzed by flow cytometry at day 8. Data are representative of three independent experiments. Means ±SD of triplicate culture wells. *, $P < 0.05$; **, $P < 0.01$ (two-tailed unpaired Student's *t*-test). **(f)** GM-DCs were incubated with AF647-OVA at 37° C for the indicated time, and incorporation of AF647-OVA in CD11c$^+$ cells (left) and frequency of AF647$^+$CD11c$^+$ cells (right) were analyzed by flow cytometry. Data are representative of three independent experiments. Mean ± SD of triplicate wells. (two-tailed unpaired Student's *t*-test). **(g, h)** T and B cells isolated from spleens and LNs of mice were stimulated with plate-bound anti-CD3 and soluble anti-IgM antibodies, respectively. Three days later, proliferation of T **(g)** and B cells **(h)** was assessed by [^{3}H]-thymidine incorporation. Data are representative of three independent experiments. Mean ±SD of triplicate culture wells. (two-tailed unpaired Student's *t*-test). **(i, j)** CD4$^+$ T cells purified from LNs and spleens were cultured under the Th1- **(i)** or Th17-differentiation conditions **(j)**. Cell differentiation was determined by flow cytometry. Data are representative of three independent experiments. Mean ±SD of triplicate culture wells. (two-tailed unpaired Student's *t*-test).

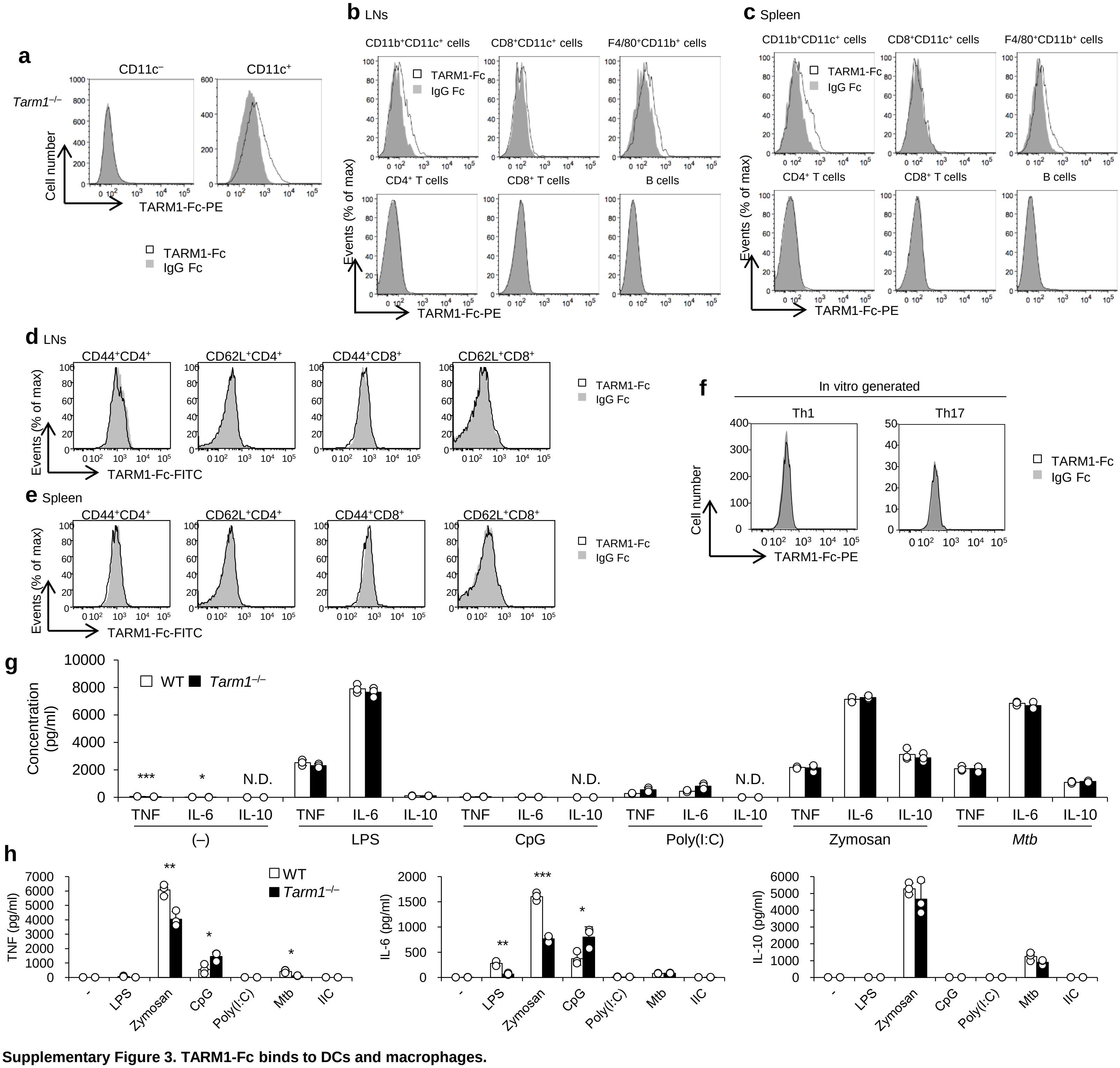

Supplementary Figure 3. TARM1-Fc binds to DCs and macrophages.
(a) Bindings of TARM1-Fc and IgG Fc to *Tarm1*$^{-/-}$ GM-DCs were analyzed by flow cytometry. Data are representative of three independent experiments. **(b, c)** Bindings of TARM1-Fc to DC ($CD11b^{+}CD11c^{+}$ and $CD8^{+}CD11c^{+}$), macrophages ($F4/80^{+}CD11b^{+}$), T cells ($CD4^{+}CD3^{+}$ and $CD8^{+}CD3^{+}$) and B cells ($B220^{+}$) of LNs **(b)** and spleen **(c)** of WT mice were analyzed by flow cytometry. IgG Fc was used as a control. Data are representative of three independent experiments. **(d, e)** Bindings of TARM1-Fc to T cells ($CD44^{+}CD4^{+}$, $CD62L^{+}CD4^{+}$, $CD44^{+}CD8^{+}$ and $CD62L^{+}CD8^{+}$) from LNs **(d)** and spleen **(e)** of CIA-induced WT mice were analyzed by flow cytometry. IgG Fc was used as a control. **(f)** Bindings of TARM1-Fc to in vitro generated Th1 and Th17 cells were analyzed by flow cytometry. IgG Fc was used as a control. Data are representative of three independent experiments. **(g)** WT and *Tarm1*$^{-/-}$ GM-DCs were stimulated with LPS (100 ng/ml), CpG (10 μM), poly(I:C) (10 μM), zymosan (100 μg/ml) and *M. tuberculosis* (Mtb; 100 μg/ml) for 24 h. Cytokine concentrations (TNF, IL-6 and IL-10) in culture supernatants were determined by flow cytometry with cytometric beads. Data are representative of three independent experiments. Mean ± SD of triplicate wells. N.D., not detected. *, $P < 0.05$; ***, $P < 0.001$ (two-tailed unpaired Student's *t*-test). **(h)** WT and *Tarm1*$^{-/-}$ BM-neutrophils were stimulated with 100 ng/ml LPS, 100 μg/ml zymosan, 10 μM CpG, 10 μM poly(I:C), 100 μg/ml *M. tuberculosis* H37 RA (Mtb) and 100 μg/ml IIC for 24 h. Cytokine concentrations (TNF, IL-6 and IL-10) of culture supernatants were determined by flow cytometry with cytometric beads. Data are representative of three independent experiments. Mean ± SD of triplicate wells. *, $P < 0.05$; **, $P < 0.01$; ***, $P < 0.001$ (two-tailed unpaired Student's *t*-test).

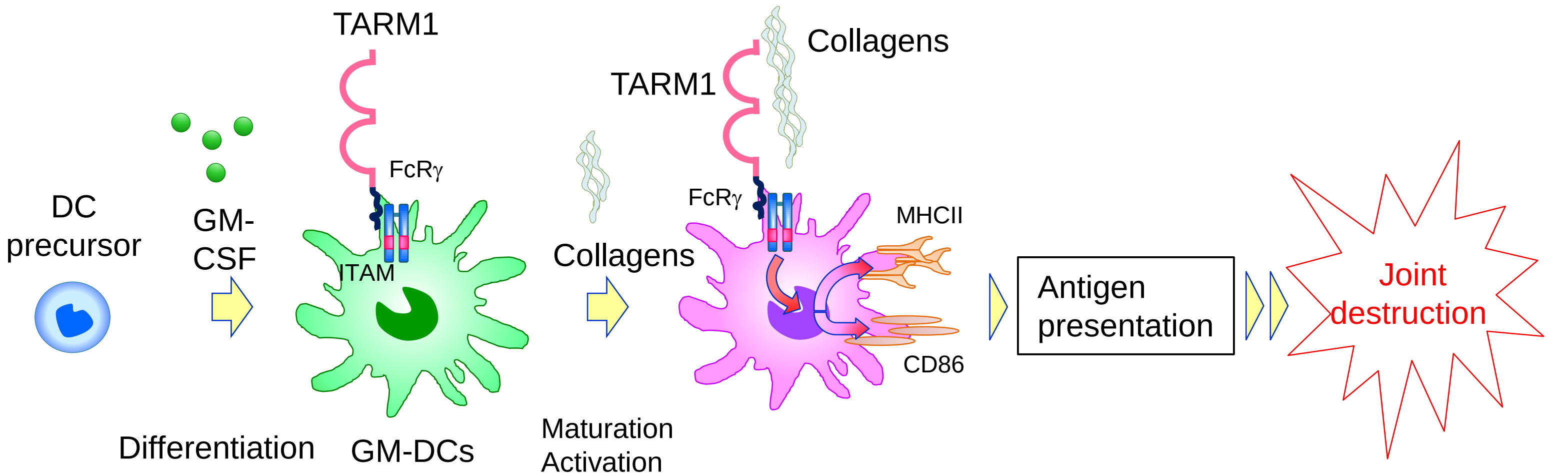

Supplementary Figure 4. The Role of TARM1 in the Development of Autoimmune Arthritis.

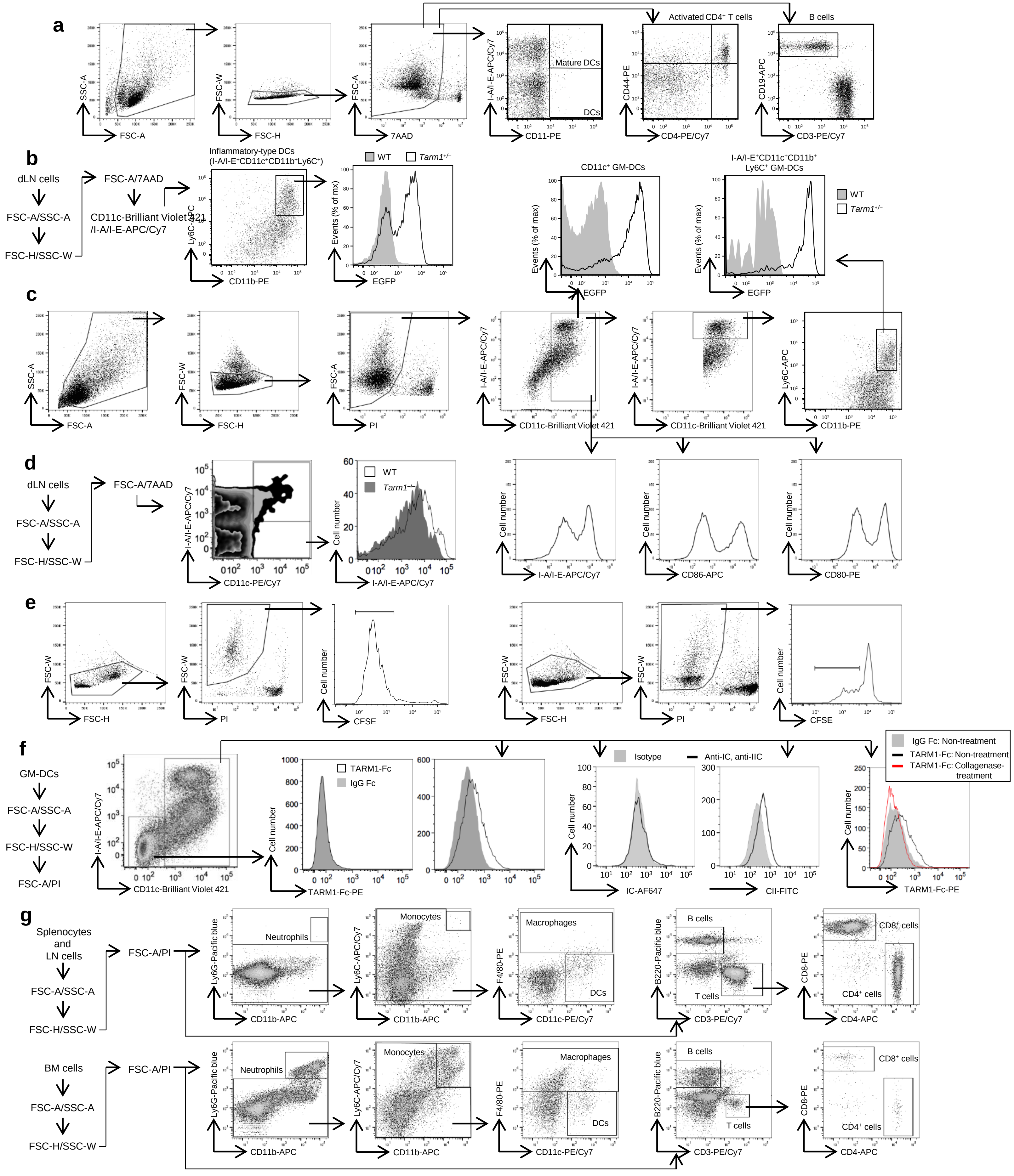

Supplementary Figure 5. Flow cytometry gating strategies for characterization of the cell populations.
(a) This gating strategy is related to Figure 1g. **(b)** This gating strategy is related to Figure 2a. **(c)** This gating strategy is related to Figure 2b, 2d, 2e, 2f, 4g, 4i, Supplementary Figure 2d and 2e. **(d)** This gating strategy is related to Figure 3e and 3f. **(e)** This gating strategy is related to Figure 3h and 3i. **(f)** This gating strategy is related to Figure 4a, 4d, 4e and Supplementary Figure 3a. **(g)** This gating strategy is related to Supplementary Figure 1h.

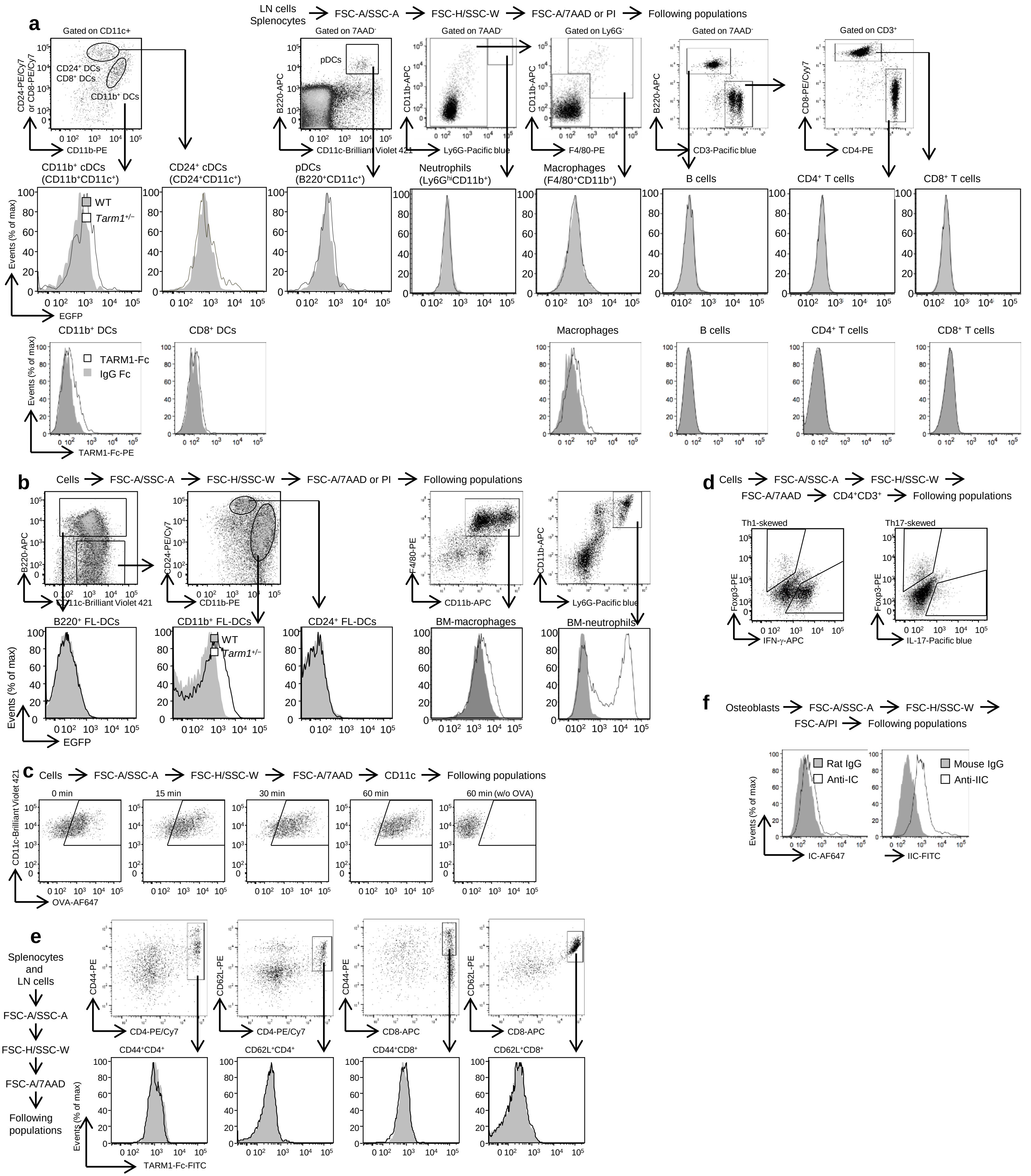

Supplementary Figure 6. Flow cytometry gating strategies for identification of the cell populations.
(a) This gating strategy is related to Supplementary Figure 2a, 3b and 3c. **(b)** This gating strategy is related to Supplementary Figure 2b. **(c)** This gating strategy is related to Supplementary Figure 2f. **(d)** This gating strategy is related to Supplementary Figure 2i, 2j and Supplementary Figure 3f. **(e)** This gating strategy is related to Supplementary Figure 3d and 3e. **(f)** Validation of anti-IC and anti-IIC antibodies to flow cytometry application. Osteoblasts were incubated with 5 μg/ml anti-IC or anti-IIC antibodies on ice for 30 min followed by 2.5 μg/ml AF647-anti-rat IgG or FITC-anti-mouse IgG on ice for 30 min. Cells were analyzed by flow cytometry. Rat IgG2a and mouse IgG were used as controls.
