# Supplementary material for: TARM1 contributes to development of arthritis by activating dendritic cells through recognition of collagens
Source: Nat Commun. 2021 Jan 4;12:94. doi: 10.1038/s41467-020-20307-9 (PMC7782728; doi:10.1038/s41467-020-20307-9)
Supplement: Supplementary file 3 — Descriptions of Additional Supplementary Files [file 41467_2020_20307_MOESM3_ESM.pdf]

Descriptions of Additional Supplementary Files

Supplementary Data 1

Description: A list of gene expressions of WT and Tarm1-/- GM-DCs used for DNA microarray in this study.
